# Positive effects of grasping virtual objects on memory for novel words in a second language

**DOI:** 10.1038/s41598-020-67539-9

**Published:** 2020-07-01

**Authors:** M. Macedonia, A. E. Lehner, C. Repetto

**Affiliations:** 1 Department of Information Engineering, Johannes Kepler University, Linz, Austria; 2 Linz Center of Mechatronics GmbH, Johannes Kepler University, Linz, Austria; 3 Lise Meitner Research Group “Cognition and Plasticity”, Max Planck Institute for Human Cognitive and Brain Sciences, Leipzig, Germany; 4 Department of Linguistics, University of Vienna, Vienna, Austria; 5 Department of Psychology, Università Cattolica del Sacro Cuore, Milan, Italy

**Keywords:** Cortex, Human behaviour

## Abstract

Theories of embodied cognition describe language processing and representation as inherently connected to the sensorimotor experiences collected during acquisition. While children grasp their world, collect bodily experiences and name them, in second language (L2), students learn bilingual word lists. Experimental evidence shows that embodiment by mean of gestures enhances memory for words in L2. However, no study has been conducted on the effects of grasping in L2. In a virtual scenario, we trained 46 participants on 18 two- and three-syllabic words of Vimmi, an artificial corpus created for experimental purposes. The words were assigned concrete meanings of graspable objects. Six words were learned audio-visually, by reading the words projected on the wall and by hearing them. Another 6 words were trained by observation of virtual objects. Another 6 words were learned by observation and additional grasping the virtual objects. Thereafter participants were subministered free, cued recall, and reaction time tests in order to assess the word retention and the word recognition. After 30 days, the recall tests were repeated remotely to assess the memory in the long term. The results show that grasping of virtual objects can lead to superior memory performance and to lower reaction times during recognition.

## Introduction

Embodied cognition holds that cognitive processes are rooted in the body’s interaction with the world surrounding us^1–4^. Accordingly, language, as a cognitive ability, is grounded in our sensorimotor systems^5,6^ and the representation of words is tightly bound to the bodily experiences that we collect when acquiring them^7–10^. In fact, during language acquisition, infants grasp and manipulate objects and all sort of things they can catch. By doing so, infants collect multiple sensorimotor experiences within their environment. Infants are *not* taught to grasp, drop, smell, taste food or objects coming into their hands. They simply do it and they naturally learn shape, weight, odour and whether a fruit is sweet or sour by biting it and sipping its juice, by interacting with it^11^. At a certain time point of their cognitive and linguistic development, children associate sequences of phonemes articulated by the caregivers to the fruit, i.e. children learn how to name it^12,13^. Accordingly, on the base of the collected experiences, objects have different sensorimotor representations. In the brain, a lemon will be represented differently than a strawberry, despite the fact that linguistically they are both categorized as fruits. The sensorimotor interaction that both fruits allow differs, so the neural representations will be different^14^.

Neuroimaging experiments show that the motor system is involved in word processing^15–18^. Scientists stimulate the sensorimotor networks created during language acquisition by presenting the “label” to the experience, the word, acoustically or visually. While resonating upon stimulation, the word evokes motor responses that can be topographically well defined^19^. They can detect even the single limbs engaged during the concept acquisition^15^. Similarly, these networks also include in their structure odour^20^, taste^21^, colour^22^, and all sensory and motor-related features connected to the experiences that the person has collected. Words are not abstract entities in the brain’s language^23^. Words are labels to experience related sensorimotor networks^24^.

Evolutionary theories have described grasping as a precursor of speech^11^. In the “gesture first” hypothesis, the grasping of objects, their manipulation and gestural abstraction of motor acts (mimes) is described as having given birth to a protolanguage. It first combined both gestural and vocalized communication. Later on, the shift from gestures to vocalizations might have come with the necessity to communicate on more complex and abstract contents. Corballis^25,26^ points to language as a gestural system—spoken language being a component of it—that has evolved within the mirror neuron system in the primate brain^27^. This “system”, i.e., a large brain network, includes also Broca’s area, the language core region^28^. In a recent review article^29^, Kendon challenges the gesture first hypothesis by questioning the evolutionary switch from gestures to speech. Taking into consideration that the vocal apparatus is there in humans, Kendon suggests instead the oral-aural hypothesis. Accordingly, language grew in complexity with the complexity of social organisation. Although science might never be able to assess whether gestures came before oral communication, or if gesture developed together with it,—as addressed by Kendon^29^, one thing is sure: motor acts scaffold language and language acquisition.

The way we acquire information has an impact on memory and on how easily we can retrieve it. When we grasp things, we represent them in in our brains^30^. In a study by Madan and Singhal^31^, participants encoded concrete words in L1 related to objects with high vs. low manipulability as *camera* and *table* while accomplishing judgement tasks as word length, functionality, etc. The results of the free recall test showed better memory performance for high-manipulability words, i.e., for words that are linked to stronger motor-related processing. Hence, the more intensively we interact with things, the better their representations will be grounded. Thus, it does not surprise that neuroimaging studies on words for tools or instruments show stronger activity in motor brain areas than objects that are less manipulable^32,33^. In the brain, grasping and object manipulation create strong sensorimotor networks connected to the concepts and hence to the words. The proof to this comes from experiments with repetitive transcranial magnetic stimulation (rTMS). If motor networks are disturbed by means of rTMS, reaction speed in responses is slowed down for words that are action related. This indicates that the motor component is integral part of the word representation^34,35^.

Sensorimotor networks modulate memory for words, i.e. they make verbal information resistant against decay. In fact, concrete words, i.e. words allowing multisensory and sensorimotor representation are remembered better than abstract words^36,37^. Research accounted first for better word or phrase retrievability by connecting a motor component to the word’s representation. Accordingly, if a word or a phrase is additionally encoded with action, a motor “trace” would improve its memorability^38^. This effect has been named enactment^39^ or self-performed task effect^40^. A number of experiments on the enactment effect have shown that it is applicable to memory for words and phrases in different context and presented to different populations^41–43^.

Despite the fact that in the last decades a multitude of methods have been developed^44^, vocabulary learning in L2 mainly takes place by means of listening and comprehension, and writing activities^45,46^. Also (paper-based^47^ and digital^48^) flash cards are used to make vocabulary items more durable in memory^49^. However, basically, in L2 classes, learners sit, listen and read. This is possibly due to the fact that in twentieth century linguistics, language has been described as symbolic and amodal^23^, as a phenomenon of the mind (with no connection to the body). Considering the cumulative evidence in L1 for the strong link between sensorimotor experience and representation of concepts, we reason that vocabulary training explicitly should connect novel words in L2 with these sensorimotor experiences.

In L2 instruction, vocabulary learning has been related to activities focussing on semantic relationships among words within a text, and to imagery^44^. Embodied activities as the use of gestures, instead, have been rare. In 1995, Quinn-Allen^50^ conducted one of the first experiments on the benefit of emblematic gestures accompanying phrases in L2. Other studies followed and demonstrated that gestures accompanying novel words in L2 lead to superior memory performance if compared to reading and listening (please see for reviews^51,52^) but also to pictures related to the words^53^. This has been shown for concrete but also for abstract words in the short and the long term in experimental settings^54^ but also during live lessons^55^ for actions, iconic and symbolic and, more recently, also for idiosyncratic gestures^56^. These studies have demonstrated that better retention and slower decay of words is due to the creation of large sensorimotor networks connected to the novel phoneme sequence^57^. Furthermore, there is evidence that L2 words learned with gestures and actions have access to brain areas related to declarative but—due to the motor input—also to procedural memory^58^.

It is relevant to distinguish between gestures and motor acts. Not any movement can support memory for L2 words. In fact, in L2 only congruent gestures^56^ and not semantically unrelated movements have shown positive effects on word retention^59^. Incongruent or semantically unrelated gestures paired to novel words in L2 do not lead to the same memory results as congruent gestures^57,60^. In the brain, incongruent gestures paired to novel words evoke activity in a network related to cognitive control, similar to Stroop tasks^57^. So, it is conceivable that a sensorimotor network of a gesture, connected to a L1 word, also connects to a novel word in L2 if they both are wired together by means of training. Similarly to L1 studies, also in L2 the link between sensorimotor networks and words can be disrupted by transcranial magnetic stimulation and impair language translation^61^.

Gestures have a multitude of cognitive aspects^62,63^: complexity of action sequencing, iconicity, imagery, emotional value, duration, relatedness to salient information to the speaker, pre-existing experiences, knowledge and cultural aspects of their use^63,64^. All these aspects modulate differently their impact on memory for words. In gesture studies, it is impossible to disentangle these aspects from each other. Altogether, we claim that gestures support memory for words^51,54^ but we do not know which aspect of a gesture might be the salient one, the one leading to the memory enhancement.

Grasping and manipulation can be considered a special sort of motor acts. In the infants’ world, these motor acts represent the first step of interaction with objects and things. These motor acts are directed towards the formation of sensorimotor networks: They map the physical characteristics of objects and construct a cognitive representation for the objects themselves and the related concepts^65,66^. In other words, grasping and manipulating experiences are the base to gestures.

In this study, we pose the question whether grasping virtual objects can support memory for words in L2. In a recent study, Buccino and colleagues^67^ have provided evidence that graspable nouns in L2 also modulate the motor system as words in native language (L1). Italian participants with a C1 proficiency level of English were presented with 32 stimuli consisting of photos and nouns of graspable and non-graspable objects, scrambled images and nonsense pseudowords. Participants were asked to press a key when the object/English word referred to a real object and to refrain from responding when the stimulus presented was meaningless. The results showed a main effect of object graspability with slower responses to stimuli referring to graspable objects as compared to stimuli referring to non-graspable objects. These results were in line with the results of an experiment conducted with Italian subjects who had to accomplish the same task in their native language^68^. The authors of the studies explain the slower—however unexpected—reaction times for graspable items with a possible re-enactment taking place when participants processed the stimuli. Despite these two studies, literature also has documented that the access to words with a motor component is faster than to words without motor component and accordingly that reaction times are lower. The effect has been described as pop-out effect displaying shorter reaction times for retrieval in words with motor representation^69,70^. If grasping and manipulation of objects is one of the natural “methods” that infants use to acquire the cognitive representation of concepts^71^ and therefore words^5^, we reason that learners of L2 could benefit of grasping. It is conceivable that grasping and hearing / reading novel labels to the experience might make sensorimotor networks resonate to the concept created during L1 word acquisition.

In VR, to our knowledge only a study has been conducted on novel word learning with graspable objects. Gordon and colleagues^72^ asked their subjects to grasp and manipulate six novel virtual objects either with their left or with their right hand. Thereafter, the subjects were tested on the words with a word-colour match task performed either with the hand used to grasp the virtual objects or with the other hand. Reaction times were lower when the hand used to grasp was the same as the hand involved in the response. This affordance compatibility effect was also given in two follow up experiments—to a smaller extent however—if the subjects had only watched virtual hands interacting with the objects. This is to say that sensorimotor experiences enable word representations in cognition even if the grasping is related to virtual objects.

In this study, we made use of a virtual reality (VR) scenario as a stimulation environment. We opted for VR for the reasons explained in a number of publications^73,74^. Generally, in VR subjects can access different types of multimodal, (and) social environment, and interaction mediated by the VR. Virtual worlds are ubiquitous and users have the possibility to train at any time. Users also can be provided with personalized training and learning anxiety can be reduced. Furthermore in our case, if grasping virtual objects could support word learning better than audio-visual input, i.e. reading and listening, normally accomplished with tedious lists—VR could be a vocabulary learning tool for a large number of vocabulary items, at least for graspable objects and possibly reconstruct natural processes that occur in childhood.

Taken all these considerations together, we asked

- i) whether grasping virtual objects can lead to better memory performance for L2 words than audio-visual learning, and
- ii) whether only observing the virtual objects can also benefit memory compared to reading the words and listening to them.

In our study, participants were trained in the Deep Space, a VR cave with a projection wall of 9 × 12 m at the Linz Museum Ars Electronica Center (Austria). Participants wore 3-D-glasses and experienced a full immersion in the VR. It simulated the perspective of a scuba diver on the sea ground. Participants saw oversize objects that were plunged into the water. Participants were asked to grasp the virtual objects presented singularly. We opted arbitrarily for this scenario because among the scenarios at our disposal this one was realistic and colourful with corals and fishes swimming in the reef.

Forty-six German speakers were trained on 18 words of Vimmi, an artificial corpus created for experimental purposes. Subjects learned 6 words by reading them and hearing an audio file, i.e. Audio-Visually (AV), 6 words by enriching the AV-input with the corresponding image of the Object (AVO) and 6 words by additionally having the subjects grasp the virtual Objects (AVGO). In a pilot phase of the experiment, in the AV condition, participants asked expressly to sit down and to reduce the stimulation time. In fact, only reading the words and hearing the audio file takes less than observing/and grasping the object plunged into the underwater landscape. So, we allowed participants to sit down and we reduced the stimulation time. By doing this, we tried to make them feel comfortable and keep their cooperation high, considering that many of them were elderly subjects. At the same time, we are aware that these differences may have an effect on the learning condition.

The training lasted approx. 80 min. Thereafter, we subministered different tests to assess word retention in the short and long term, in both languages (free recall German, Vimmi and paired free recall, cued recall from German into Vimmi and viceversa), finally a word and an image recognition test. During breaks, participants were allowed to talk to each other, to move around and to use their smartphones.

We hypothesized that.

1. Grasping the virtual objects leads to better memory performance than reading and hearing the words to them, and observing the objects, and
2. Reaction times in word recognition are lower for words that have been encoded by means of grasping.

## Results

The recall tests were scored assigning a value of 1 for each correct response, and a value of 0 in case of incorrect response or omission. Therefore, for each test the total score ranged from 0 to 18. For the recognition tests, the incorrect responses were identified and deleted (6% of all the trials); RTs were considered only for items correctly recognized.

**Figure 1:**
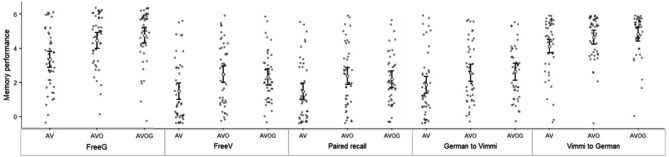
Memory performance in the Immediate recall tests. *FreeG* free recall in German, *FreeV* Free recall in Vimmi, *Paired recall* free paired recall, *German to Vimmi* Cued recall from German to Vimmi, *Vimmi to German* cued recall from Vimmi to German. Error bars indicate 1 standard deviation.

**Figure 2:**
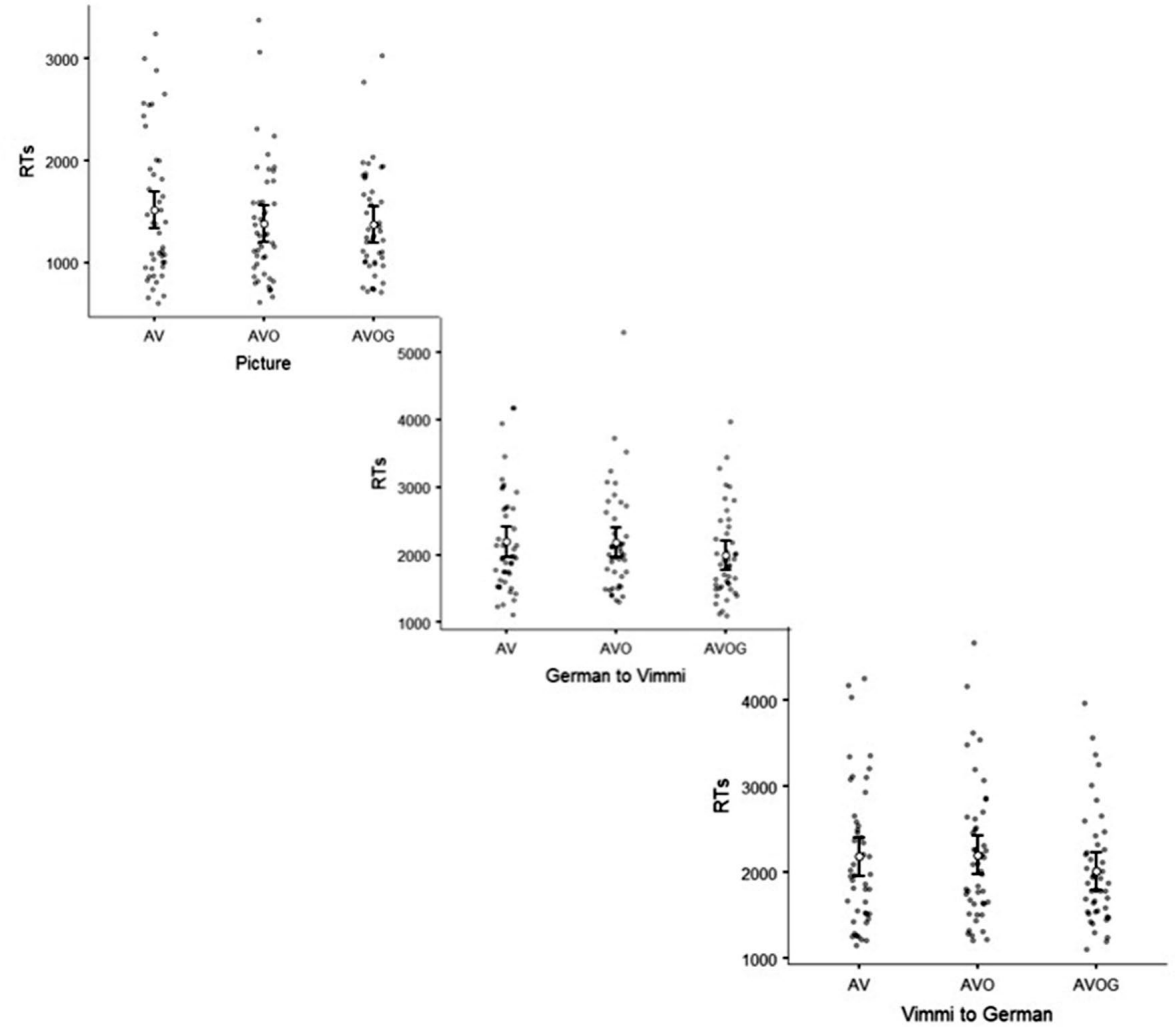
Reaction times in recognition tests. Error bars indicate 1 standard deviation.

**Figure 3:**
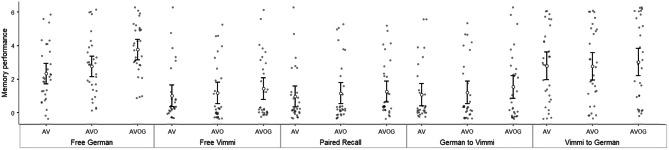
Memory performance in the Delayed recall tests. *FreeG* free recall in German, *FreeV* Free recall in Vimmi, *Paired recall* free paired recall, *German to Vimmi* Cued recall from German to Vimmi, *Vimmi to German* cued recall from Vimmi to German. Error bars indicate 1 standard deviation.

Table 1 reports descriptive data for all the variables included in the analysis, and averaged by subject. The same data were plotted grouping together the Immediate Recall tests (Fig. 1), the Recognition tests (Fig. 2), and the Delayed Recall tests (Fig. 3).

**Table 1:** Descriptive statistics.

		**N**	**Min**	**Max**	**Mean**	**St. Dev**
Immediate recall						
Free recall German	AV	46	0	6	3.35	1.69
	AVO	46	0	6	4.46	1.52
	AVOG	46	0	6	4.76	1.54
Free recall Vimmi	AV	46	0	6	1.61	1.86
	AVO	46	0	6	2.67	1.94
	AVOG	46	0	6	2.50	1.49
Paired free recall	AV	46	0	6	1.59	1.83
	AVO	46	0	6	2.54	1.94
	AVOG	46	0	6	2.35	1.55
German to Vimmi	AV	46	0	6	2.00	1.89
	AVO	46	0	6	2.74	1.81
	AVOG	46	0	6	2.80	1.63
Vimmi to German	AV	46	0	6	4.48	1.76
	AVO	46	0	6	5.02	1.31
	AVOG	46	0	6	5.22	1.30
Recognition						
Picture	AV	46	607.17	3,241	1516.29	699.58
	AVO	46	616.8	3,381.5	1,384.37	590.80
	AVOG	46	709.67	3,026	1,369.85	520.19
German to Vimmi	AV	46	1,113.17	4,179.83	2,215.09	776.9
	AVO	46	1,308.67	5,303	2,187.79	764.55
	AVOG	46	1,104.5	3,975.5	1988.51	651.44
Vimmi to German	AV	46	1,156.5	4,257.2	2,187.79	821.97
	AVO	46	1,214	4,675.4	2,202.61	800.45
	AVOG	46	1,112	3,970.67	2015.88	645.18
Delayed recall						
Free recall German	AV	30	0	6	2.33	1.67
	AVO	30	0	6	2.77	1.81
	AVOG	30	1	6	3.77	1.5
Free recall Vimmi	AV	30	0	6	1	1.66
	AVO	30	0	5	1.17	1.80
	AVOG	30	0	6	1.43	1.91
Paired Free recall	AV	30	0	6	0.97	1.61
	AVO	30	0	5	1.17	1.86
	AVOG	30	0	5	1.27	1.70
German to Vimmi	AV	30	0	6	1.07	1.82
	AVO	30	0	5	1.20	1.85
	AVOG	30	0	6	1.53	1.91
Vimmi to German	AV	30	0	6	2.83	2.20
	AVO	30	0	6	2.8	2.24
	AVOG	30	0	6	3.07	2.38

*Min* minimum value, *Max* maximum value, *St. Dev.* standard deviation.

To evaluate the efficacy of grasping on word retention and word recognition we applied Linear Mixed Models, which allow to account for two sources of random variability, i.e., participants and words^75,76^. For all the recall tests, considering that our dependent variable (accuracy) was binomial (1 = hit; 0 = fail), we chose the Generalized Linear Mixed Model (GLMM) approach. For the recognition test, as the dependent variable was continuous (RTs), we applied the standard Linear Mixed Models (LMM). As predictor we considered one factor within subjects at three levels (Encoding: AV vs AVO vs AVOG). The model included the conditions AV and AVO calculated against the reference condition AVOG. Random intercepts were considered for both participants (s) and words (w). The function representing the final model is the following (in brackets the random components):

$$ {\text{y}}_{{{\text{ws}}}} = \, \left( {{\text{u}}_{{0{\text{s}}}} + {\text{ u}}_{{{\text{w}}0}} } \right) \, + \gamma_{00} + {\text{ b}}_{{1}} *{\text{AV }} + {\text{ b}}_{{2}} *{\text{AVO }} + {\text{ e}}_{{{\text{ws}}}} . $$

Tables 2 and 3 summarize estimated parameters for GLMM and LMM respectively.

**Table 2:** Model summary of the GLMM.

	**Parameters**	**Fixed effects**			**Random effects**	
					**By subjects**	**By items**
		**Exp (coefficient)**	**CI**	**t**	**σ**^**2**^	**σ**^**2**^
Immediate recall						
Free German	Intercept	7.59	4.82–11.095	8.76***	0.42*	0.19
	AV	0.17	0.11–0.27	− 7.85***	–	–
	AVO	0.42	0.26–0.66	− 3.78***	–	–
Free Vimmi	Intercept	0.67	0.41–1.01	− 1.6	1.32***	0.23
	AV	0.46	0.31–0.68	− 3.82***	–	–
	AVO	1.11	0.76–1.62	0.53	–	–
Paired recall	Intercept	0.65	0.4–1.05	− 1.76	1.38***	1.19
	AV	0.39	0.26–0.58	− 4.54***	–	–
	AVO	0.93	0.64–1.37	− 0.34	–	–
German to Vimmi	Intercept	0.72	0.43–1.19	− 1.28	1.42***	0.32*
	AV	0.92	0.63–1.34	− 0.44	–	–
	AVO	0.95	0.65–1.4	− 0.25	–	–
Vimmi to German	Intercept	7.89	4.27–14.57	6.6***	2.13**	0.23
	AV	0.82	0.51–1.35	− 0.76	–	–
	AVO	0.93	0.57–1.53	− 0.28	–	–
Delayed recall						
Free German	Intercept	1.85	1.03–3.31	2.09*	1.08*	0.4*
	AV	0.32	0.2–0.52	− 4.65***	–	–
	AVO	0.47	0.3–0.75	− 3.13**	–	–
Free Vimmi	Intercept	0.17	0.07–0.41	− 3.97***	3.16**	0.64
	AV	0.55	0.29–1.08	− 1.75	–	–
	AVO	0.78	0.4–1.51	− 0.74	–	–
Paired recall	Intercept	0.14	0.06–0.33	− 4.38***	3.3**	0.56
	AV	0.63	0.31–1.23	− 1.36	–	–
	AVO	0.92	0.47–1.81	− 0.22	–	–
German to Vimmi	Intercept	0.21	0.09–0.5	− 3.58***	3.26**	0.4
	AV	0.54	0.26–1	− 2.06	–	–
	AVO	0.64	0.33–1.22	− 1.35	–	–
Vimmi to German	Intercept	1.17	0.47–2.94	0.35	4.37**	0.47
	AV	0.82	0.47–1.42	− 0.7	–	–
	AVO	0.83	0.48–1.44	− 0.67	–	–

*p < .05; **p < .005; ***p < .001.

**Table 3:** Model summary of the LMM.

	**Parameters**	**Fixed effects**		**Random effects**			
				**By subjects**		**By items**	
		**Estimate**	**t**	**Estimate**	**Wald** **Z**	**Estimate**	**Wald Z**
Recognition							
Picture	Intercept	1,220.97	19.72***	114,796.76	18.23***	5,065.5	1.3
	AV	103.92	2.24	–		–	
	AVO	38.25	0.83	–		–	
German to Vimmi	Intercept	2012.18	18.752***	387,847.45	18.86***	18,087.4	1.75
	AV	146.36	2.25*	–		–	
	AVO	184.62	2.89**	–		–	
Vimmi to German	Intercept	2019.24	16.94***	396,354.54	4.31***	48,639.82	2.19*
	AV	156.67	2.07*	–		–	
	AVO	174.08	2.3*	–		–	

*p < .05; **p < .005; ***p < .001.

### Immediate testing

#### Recall tests

According to the model, memory performance varied random across participants (u_0s_) in all the tests; however, it varied random across words (u_w0_) only in the cued recall from German to Vimmi and in the recognition task from Vimmi to German. Nevertheless, after controlling for the random factors, we found that in the *Free German* test the words encoded in the AVOG condition were better remembered, compared to those learned in both AV and AVO conditions. In the *Free Vimmi* and in the *Free Paired Recall* tests, the AVOG condition appeared superior only to the AV but not to the AVO condition. Furthermore, in both *Cued Recall tests* (from German to Vimmi and from Vimmi to German), the AVOG condition did not yield better performance than either AV or AVO conditions.

#### Recognition tests

In the *recognition tests*, participants were faster in recognizing words encoded with grasping (AVOG) than those encoded audiovisually (AV) or with picture (AVO), in both the German to Vimmi and Vimmi to German tests. In the *Picture recognition task*, words encoded in AVOG condition were not recognized faster than those encoded in the other conditions.

#### Delayed testing

Models evidenced that memory performance varied random across participants (u_0s_) in all the tests and across words (u_w0_) in the Free German test.

After controlling for the random effects, even after one months from the training, in the *Free Recall German* test words were better remembered if originally encoded with grasping than with picture and audiovisually. However, in all other tests (Free Vimmi, Paired Recall, cued recall from German to Vimmi and Cued Recall from Vimmi to German) no significant differences were detected from AVOG to the other conditions.

## Discussion

In this study, we asked whether grasping virtual objects (AVOG) can lead to better memory performance for L2 words than audio-visual learning (AV) and whether observing the virtual objects (AVO) can also benefit memory compared to reading the words and listening to them.

Also, we asked whether German and Vimmi words learned with sensorimotor enrichment are recognized faster than words learned in the AV and AVO conditions.

To answer these questions, after training, we administered recall and recognition tests. All tests took place immediately after training in order to verify the efficacy of the training in the short term, and recall tests were repeated approximately 30 days after training for the long term.

Recall tests measure the overall capacity to remember linguistic items (German and Vimmi free recall), the capacity to recall the word in both languages (paired free recall) and the capacity to precisely recall words from one language to the other (cued recall). This is to say that recall tests measure the capacity to use words actively, in order to name things and later to use the words in sentences. Recognition tests, instead, are an indicator for the skill to catch words when spoken/written and therefore understand L2 passively when interlocutors talk. L2-learners need both: active and passive ability to retrieve words in order to interact with other speakers.

We hypothesized that grasping of virtual objects (AVOG) would lead to better recall performance than observing the objects (AVO) and than reading the words (for objects) and hearing them (AV). This because AV encoding is a shallow way to encode information ^77,78^ and makes it decay fast as if compared to pictures^53^ or with embodied activities like gestures^52^. Also, we hypothesized that AVOG training would make word recognition faster than AV and AVO training.

In **word retrieval**, the statistical analyses confirmed our hypothesis in the German free recall. In the Vimmi free recall, the difference was significant between words encoded in the AVOG and AV conditions. These results mirror other studies with embodiment by means of gestures that had trainings of the duration of an hour^79,80^ or other studies in the first phases of the training^54^. After one hour training, subjects can remember a certain number of concepts and words in their native language^81^. The same performance cannot however be expected in L2 being the task more challenging. In fact, words in L2 consist of phonemes, phoneme sequences, and chunks that are unfamiliar and therefore need a number of repetitions and consolidation to be stored. However, considering the short training, the enhancement in memory performance between AVOG and AV is neat and proves the efficacy of grasping the virtual objects. The same considerations can be applied to the paired free recall. Cued recall tests in both directions (Vimmi to German and viceversa) did not yield significant differences in performance among conditions. This is in line with other studies with embodiment by means of gestures, in which paired recall tests could be mastered only after longer trainings, i.e., after 10 to 12 h^82^.

Considering the short duration of the training and compared to a similar study with gestures^80^, grasping yielded better results than gestures. This can possibly be attributed to the reactivation of strong sensorimotor networks^66,78^ created during infancy for shape, visual, and other features. It is speculative but these pre-existing networks might work as an attractor for the novel phoneme sequence as described by Hopfield^83^ in his model of artificial networks accounting for associative memory. Instead, gestures representing an abstraction of certain sensorimotor components of a concept’s shape, function, etc. might not exert this function as basic grasping and manipulation networks do. For example, the gesture for the concept “house” can be the converging arms hold together to reproduce the shape of a roof, a component of the house but not the house itself. Hence, gestures are not as immediate as grasping. Gestures might therefore cause more cognitive effort and be less efficient in storing verbal information. Further empirical research is however necessary to elucidate the different impact of grasping and gestures on memory for words in L2.

Furthermore, if we consider the short duration of the training related to the results, and if we compare it to the studies with gestures, we reason that grasping might be efficient because of heightened attention due the context in which the training took place. The studies with gestures were conducted with videos of an actress performing them. The present study, instead, took place in the Deep Space of the Ars Electronica Center in Linz (Austria). The Deep Space is a VR cave with an impressive projection wall of 9 × 12 m. Participants had an immersion experience similar to the one of a scuba diver observing oversize objects that were plunged into the water. The training was thus very unusual to subjects learning vocabulary items in a foreign language. This environment might have triggered a bizarreness effect^84^ with a positive effect on retention.

In **word recognition**, the results confirmed our hypothesis: German and Vimmi words learned with sensorimotor enrichment, are recognized significantly faster than words learned in the AV and AVO conditions. This is to say that sensorimotor enrichment has a positive impact on the speed of processing within the word network and is in line with other studies describing faster processing, i.e. pop out effect, for words with sensorimotor enrichment^69,70^. In the past, a number of studies have documented the language-to-action link and its related speed of processing depending on the limbs involved^85^, being the activation of limbs differentially connected (and inhibited) to words^86^. Thus, grasping virtual objects in order to remember the words in L2 makes learners recognise the words faster.

It is interesting to note here that the age of the participants was quite heterogeneous due to the participation of museum visitors in the experiment. On average, they were 36.61 ys old with a SD of 15.95 ys. This age structure is unusual in learning experiments and older age is related to declining memory capacity^87^. However, this age structure did not compromise the overall experiment results indicating that AVOG is a strong tool to learn novel verbal information also in a heterogeneous population with elder subjects. If further research can demonstrate that memory decline in elder learners could be compensated by grasping activities in L2 word learning, grasping should be considered as an effective strategy in L2 instruction.

There is evidence that interacting with real objects enhances learning of L2 words^88^. In our study however, we opted for virtual reality instead of a naturalistic environment with real objects or videos with the objects to be virtually grasped. The reasons leading to this are the following. First, we wanted to realize a novel kind of learning environment that makes learning less tedious than classroom ambience. Second, we wanted to have 3D objects in order to simulate interaction with the object near to real perception. Third, the VR underwater landscape allowed full control for the time of exposure and interaction with the virtual objects that were plunged into the scenario at the same time for all subjects and had all the same dimensions. This also allowed us to train multiple subjects simultaneously. Forth, this experiment took place in a room with a huge projection wall, but VR can be implemented in smartphones and VR-cardboards. Accordingly, VR as a training tool for language learning can be individualized according to the user’s preferences and needs and more importantly, VR can be used everywhere and anytime^89^. Also, VR has a huge potential to facilitate language learning also for persons that for physical, financial and geographical reasons cannot access live instruction^90^.

In a recent article, Kühne and Gianelli^91^ ask whether embodied cognition is bilingual and review empirical research in L1, and the scarce literature in L2. The authors come to the conclusion that studies in L1 and L2 are not comparable for a number of reasons and that the question remains unanswered. There is evidence that if L2 has reached a certain expertise, also L2 can trigger motor responses^92^. Also, there is evidence that novel pseudowords can be embodied. In fact, in studies with fMRI, the motor system responds to verbal stimulation^57^ but also other areas involved in sensory encoding come into play if verbal information has been encoded with an embodied learning strategy^93^.

Taken together, the results of our study show that if words in L2 are learned by grasping virtual objects in a VR environment, their memorability is enhanced as well as their recognition. Learners that learn by means of sensorimotor enrichment can thus retrieve more words to build sentences and can understand them better while listening to other interlocutors. In other words, our study provides evidence for grasping as a procedure to enhance audio-visual learning of words in L2. Grasping recreates sensorimotor experiences in the classroom and simulates L1 learning. VR makes grasping reproducible and ubiquitous without real objects and can embed embodied learning of L2.

## Methods

### Participants

Forty-six German natives took part in the experiment (mean age 36.61 years, SD = 15.59, 19 males; mean education 14.8 years, SD = 2.75). They were recruited from a Linz University database, by advertisements at the University, through personal contacts, and by the database of the museum Ars Electronica Center (www.aec.at) where the experiment took place. Participants had normal or corrected-to-normal vision and no history of neurological and psychiatric diseases. All of them showed normal working memory abilities compared to the reference population, as assessed by the Digit Span Test^94^ (forward and backward versions). The study was conducted in compliance with the Helsinki Declaration of 1975, as revised in 2008. Local Ethics Committee (University of Linz) approval was obtained; all participants gave written informed consent prior to testing and received an entry-voucher for the AEC as compensation. Informed consent was collected also for publication of participants’ pictures within Open Access Scientific Journals.

### Materials

The stimulus material comprised 18 vocabulary items of Vimmi an artificial corpus designed for research purposes in order to avoid associations with participants’ native or foreign languages^57^. In fact, association among similar items is at the base of adult learning. Novel words should exclude the possibility of association^95^. Vimmi conforms with Italian phonotactics rules, i.e., it sounds Italian but it is not. A Perl script randomly generated phoneme sequences conforming with Italian phonotactics rules. The script controlled tautologies in syllable occurrence and frequency of sounds. Phoneme strings that might have sounded peculiar to German speaking subjects or that might have raised associations with words in languages that participants had learned before were removed manually. By creating the artificial corpus, we controlled for a number of factors that may have an influence on memory. Out of the corpus, we used 9 two-syllabic and 9 three-syllabic items. Word length, number of syllables and frequency of use of the items were equally distributed across learning conditions. The items were also controlled for parallel occurrence of initial or final phonemes within word pairs. Vimmi items were arbitrarily paired with German translation equivalents, concrete nouns denominating graspable objects. German words were selected that did not trigger sound, smell or taste related to their semantics. The complete set of items is reported in Table 4.

**Table 4:** Items used for the training.

**Vimmi**	**German**	**English**
Bofe	Computer	Computer
Wasute	Spiegel	Mirror
Toze	Brille	Eyeglasses
Mebeti	Rucksack	Backpack
Bekoni	Gabel	Fork
Dalo	Kleiderbügel	Hanger
Dawu	Kamera	Camera
Fapoge	Geschenk	Present
Lefa	Hammer	Hammer
Nabita	Flagge	Flag
Dotewe	Seife	Soap
Redu	Korkenzieher	Corkscrew
Igro	Handy	Mobile phone
Sokitu	Glas	(Water)glass
Dupi	Schlüssel	Key
Dizela	Stift	Pen
Boruda	Regenschirm	Umbrella
Zobu	Büroklammer	Paper-clip

In addition to the 18 word pairs, the stimulus material also included audio recordings of the Vimmi items, and 3D virtual representations of photographs of the objects. In order to exclude any possible visual influence from object colours, all photographs were converted into black and white. The stimuli (written words and virtual objects) appeared on a background representing a coloured coral reef with waving seagrass and swimming fishes.

### Training

The training took place at the Deep Space 8 K within the Ars Electronica Center in Linz, Austria. The Deep Space cave at the AEC offers two projection areas of sixteen times nine metres each, one on the wall and another on the floor, with an ultra-high resolution of 8 K for stereoscopic 3D visualizations. At the AEC Deep Space, this corresponds to a resolution of 8.192 × 4.320 pixels on each of the two projection areas, totalling more than 70 million pixels. This ultra-high definition resolution is achieved by eight Christie Boxer 4k30 Mirage 120 Hz projectors, combined with two XI-MACHINES COMPUTE CX4 High Performance Computing workstations, which equal 400 ordinary office computers. A 5.1 Surround Sound system with Kling & Freitag speakers and Sennheiser microphones delivers prime audio quality. Due to these unique properties of the AEC Deep Space, visitors can be completely immersed into cinematic, photographic or virtual sceneries. In order to experience such sceneries, 3D glasses have to be worn inside the Deep Space. For this experiment, a VR learning programme was developed with Unity 5.4 software (Unity Technologies, San Francisco, USA) by programmers from Johannes Kepler University Linz, Ars Electronica Solutions (www.aec.at/solutions) and Ars Electronica Futurelab (www.aec.at/futurelab). Devised as an app, the programme was started by the experimenter directly from the Deep Space computer system by selecting the app from the computer screen (which was also displayed on a smartphone) and by starting the programme with an XBOX 360 wireless controller (Microsoft Corporation, Redmond, USA).

Each participant was trained according to the same three training conditions. Their order was counterbalanced across participants. In the audiovisual (AV) condition, the written Vimmi and German word pair appeared on the wall, projected in a large yellow font, at the centre of the coral reef. After 1 s, the respective recording of the Vimmi word was played aloud once. The presentation of the item lasted for a total of 5 s. Participants listened to audio recordings of spoken Vimmi words and read a projected written form of the Vimmi word with its German translation equivalent juxtaposed to it. After a 4-s break with an empty coral reef, the next item followed. The second condition was the AVO condition (audiovisual + object). Here, subjects listened to the audio recording of the Vimmi items, read the Vimmi and German written word pairs and were then additionally shown the virtual object. The AVOG condition (audiovisual + object picture + grasping), comprised all the elements of the prior conditions, with an additional sensorimotor task: participants were instructed to grasp the virtual objects’ contours with both hands. Since the objects were virtual projections, participants had no haptic experience.

#### Stimulation

For the AVO and AVOG conditions, the written Vimmi and German word pair appeared, and after 1 s the Vimmi audio recording was played once. After a total of 5 s, the written words vanished. Item presentations were, up to this point, equal to the presentations in the AV condition. However, since AVO and AVOG constituted enriched learning conditions, they were also enriched timewise: in both, AVO and AVOG, the written items and the audio recordings were followed by a 10-s virtual projection of the object pictures. The projection showed the object “plunging” into the water from the top of the Deep Space screen, and landing on the coral reef ground after 1 s. There it remained for the rest of the projection time without further movements before it faded out. After the object projections disappeared, the written Vimmi and German word pairs were again displayed for 5 s at the centre of the coral reef. After the first second, the Vimmi audio recording was played once more. This was followed by a 4-s break with a void coral reef scene, before the next item appeared. Within each training condition, 6 word pairs were presented 12 times in random order.

Whereas the AV condition lasted 10 min, the AVO and AVOG conditions lasted 35 min. each. Between the conditions, participants were given two 3-min breaks. Therefore, in total, the complete training phase took around 80 min. Participants were allowed to sit during the AV condition, but were asked to stand during AVO and AVOG conditions.

**Figure 4:**
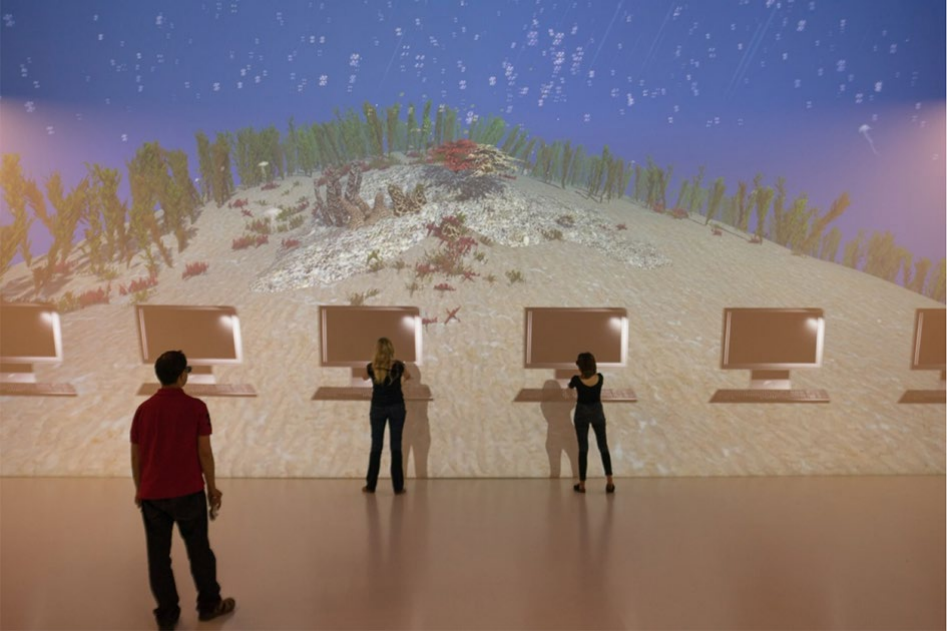
Participants during training.

Due to the size of the Deep Space, up to six participants were invited to take part in each experiment session. Throughout the experiment, each participant was positioned at a defined spot in the Deep Space, directly facing one of the six stimuli projections. These spots were marked as a white square on the floor. For each of the three learning conditions, participants were instructed to move to another defined spot (always to the second position to the left of the participant’s current position). This way, learning took place in different areas in front of the screen, left, centre, and right. This was intended to control for the possibility of a participant’s position influencing the learning process. One stimulus for each participant was projected at the same time. Therefore, six objects were displayed in a parallel line at the bottom of the screen (Fig. 4).

### Testing

After the training phase at the Deep Space, participants were given a ten-minute break and were subsequently assessed on their memory performance individually in a separate computer room.

Participants were subministered following tests: (a) In German Free Recall, the subjects were asked to write down all German items they were able to retrieve. Vimmi Free Recall followed and participants did the same for Vimmi. In the paired free recall test, participants wrote down as many word pairs as they could remember. In the cued recall Vimmi, participants were presented with a list containing all 18 German items. Participants translated as many as they could into Vimmi. Likewise, in the cued recall German, Vimmi vocabulary items had to be translated into German. The order of succession of the German and Vimmi items was randomized and different for both cued recall tests but the same for every subject. All tests had a duration of five minutes each.

Furthermore, subjects took a Recognition test with E-prime software (Psychology Software Tools, Inc., E-Prime 2.0 (2012), https://www.pstnet.com. After a brief training run to familiarize with the test, participants were presented with three subtests. In the German to Vimmi Recognition test, a German word was presented in the top centre of the screen; underneath, 3 Vimmi words were displayed in a line, at the right, centre and left. Each Vimmi option was included within a coloured text-box (grey-left; yellow-centre; light blue-right). The correct translation was always associated with 2 incorrect but plausible fillers (translations of another word learned during the training). Likewise, in the Vimmi to German Recognition test, a Vimmi word was presented and the participants had to select the correct translation out of 3 German words.

In the Picture Recognition test, object pictures were displayed as targets, and their correspondent Vimmi word had to be selected out of 3 options. Participants had to choose the right option in each recognition test by pressing the key that had the same colour of the textbox with the correct answer (“V” key was associated with grey colour, “B” with yellow, and “N” with light blue). The position of the correct answer was counterbalanced so that it was equally presented in the left, centre, and right place. RTs were recorded.

The total duration of the testing phase, i.e. for all tests, was 40 min. Thirty days after encoding, participants were contacted by email and were asked to fill an online questionnaire including the recall tests (German Free Recall, Vimmi Free Recall, Paired Recall, Cued Recall Vimmi, and Cued Recall German).

## Supplementary information

Supplementary file1 (XLSX 42 kb)

Supplementary file2 (XLSX 43 kb)

Supplementary file3 (XLSX 54 kb)

Supplementary file4 (XLSX 22 kb)

Supplementary file5 (XLSX 31 kb)

Supplementary file6 (XLSX 26 kb)

Supplementary file7 (XLSX 37 kb)
